# Co-Activation of Human Whole Blood Cells with Lipopolysaccharides and an Allergen

**DOI:** 10.3390/life13081672

**Published:** 2023-07-31

**Authors:** Svetlana V. Zubova, Ninel I. Kosyakova, Sergey V. Grachev, Isabella R. Prokhorenko

**Affiliations:** 1 Hospital of Pushchino Scientific Center, Russian Academy of Sciences, Pushchino 142290, Russia; nelia_kosiakova@mail.ru; 2 Department of Molecular Biomedicine, Institute of Basic Biological Problems, Federal Research Center “Pushchino Scientific Center for Biological Research of the Russian Academy of Sciences”, Pushchino 142290, Russia; grachevscience@gmail.com (S.V.G.); isabella03@rambler.ru (I.R.P.)

**Keywords:** lipopolysaccharides, *R. capsulatus*, Der p 2, TLR4, CD14, CD11b, cytokines, chemokines

## Abstract

The investigation of common inflammation mechanisms caused by exogenic compounds of microbial origin and allergens is one of the most important tasks in current biomedical science. The main manifestations of immune cell activation caused by pro-inflammatory agents are changes in receptor quantity on the surface of immune cells and the production of cytokines and chemokines by blood cells. The levels of expression of TLR4, CD14, and CD11b in the monocytes and neutrophils of human whole blood in response to LPS *E. coli*, Der p 2 allergen, or their combination reflect different functional activities in these cells, while the composition and amount of produced cytokines reflect the biological activity of the studied agonists. The activity of Der p 2 allergen in ex vivo experiments on whole blood samples is significantly lower compared with its activity in vitro in isolated PBMC cells, which should be taken into account when transferring the results obtained for isolated cells to whole blood cells. LPS *R. capsulatus* PG significantly decreases the synthesis of MyD88-dependent NF-κB-regulated cytokines activated by LPS *E. coli*, Der p 2, or their combination. This indirectly indicates the general mechanisms of cell activation caused by these structures and the unified mechanism of the protective action of LPS *R. capsulatus* PG against both endotoxin and a combination of endotoxin and the allergen.

## 1. Introduction

The investigation of common inflammation mechanisms caused by exogenic compounds of microbial origin and allergens is one of the most important tasks in current biomedical science. Interactions between innate immune system cells and various bacterial components such as Gram-negative bacterial lipopolysaccharides (LPS) or with the protein structures of environmental allergens are the basis for their recognition by cell receptors and the activation of inflammatory responses. Chronic allergic reactions might be caused or exacerbated by infectious agents [1]. The most distributed year-long sources of allergens and important causes of perennial allergic rhinitis and allergic asthma are house dust mites (HDMs), belonging to the genus *Dermatophagoides* [2]. HDMs are widely distributed in the environment, and they are often contaminated with endotoxins [3].

The TLR4/MD-2 receptor complex recognizes LPS and activates intracellular signaling pathways, which results in the transfer of NF-κB transcription factor from the cytoplasm to the nucleus [4]. CD14 is a receptor that carries LPS to TLR4/MD-2 [5]. Leukocyte β2-integrin CD11b activated by endotoxins is able to affect numerous intracellular signaling pathways by regulating the intensity of TLR4-induced immune responses [6].

The most important factor mediating LPS-dependent signaling with TR4 is the myeloid differentiation protein MD-2, which forms a receptor complex with TLR4 [7]. TLR4/MD-2 is a receptor complex recognizing, along with endotoxins, the majority of clinically important protein allergens, including HDM [8]. One of the major protein allergens in HDM extracts is the Der p 2 protein, which has a significant structural similarity to MD-2. Der p 2 is capable of carrying LPS to TLR4 in the absence of MD-2 [9]. Der p 2 was detected as a member of the MD-2 protein family, binding the domains of lipids recognized by MD-2 [2]. Der p 2 contains a hydrophobic pocket capable of binding LPS and potentially binding to lipids other than LPS. The activation of immune cells by the Der p 2 allergen might occur via TLR4-dependent and TLR4-independent mechanisms [10,11]. Being a structural and functional homolog of MD-2, this allergen can activate the blood cells to cytokine synthesis itself and increase susceptibility to allergens. A study on transfected HEK MD-2293FT cells showed that, similar to LPS, Der p 2 is able to induce the dimerization of TLR4 and the activation of intracellular signaling pathways, leading to the synthesis of pro-inflammatory cytokines and chemokines, and it can increase LPS-induced TLR4-dependent responses [3]. Der p 2 causes the elevated secretion of IL-6 and IL-8 cytokines by THP-1 monocytes and B-lymphocytes via the activation of TLR4 and MAPK [12]. LPS and Der p 2 demonstrated synergistic effects on IL-8 and IL-6 produced by BEAS2B lung cell lines. A study on peripheral blood mononuclear cells (PBMC) showed that recombinant Der p 2 activates TNF-α in a dose-dependent manner. It was demonstrated that differences between the contributions of kinases depend on the activating agent (protein allergen and/or LPS): MAPK- and PI3K-dependent signaling in PBMC cytokine synthesis regulation is most pronounced in response to their activation with Der p 2 [13]. Earlier, we showed that Der p 2 can increase the response of human whole blood cells to LPS by inducing the additional expression of LPS-carrying protein sMD-2 [14].

Toxic LPS triggers innate immune responses depending on the composition of lipid A, particularly on the number and structure of the fatty acids of which it consists. LPS from the phototrophic bacterium *Rhodobacter capsulatus* PG is an antagonist of endotoxins, suppressing the promotion of human blood cells to release a broad spectrum of pro-inflammatory cytokines [15,16,17]. This demonstrates the potential ability of LPS *R. capsulatus* PG to block signals from TLR4 in response to LPS and/or the allergen.

The goal of the work was to examine the ability of Der p 2 and its combined action with LPS to promote human whole blood cells to express surface receptors (TLR4, CD14, and CD11b) and synthesize pro-inflammatory cytokines and chemokines (TNF-α, IL-1β, IFN-γ, IL-8). The cells were activated using recombinant Der p 2 protein, toxic LPS *E. coli*, non-toxic LPS *R. capsulatus* PG, or their combinations.

## 2. Materials and Methods

This research was carried out on the heparinized peripheral blood of healthy volunteers of both sexes (age, 25–30 years). All examinees provided written consent to participate in the study after being informed of the procedures. The research protocol corresponds to the World Medical Association Declaration of Helsinki (2013) and was approved by the Local Ethics Committee of the Hospital of Pushchino Scientific Center, protocol number 9 from 12 July 2017. The criteria for blood collection for the experiment are concordant with the requirements for blood donation: the absence of innate diseases; a medical certificate with information on all the diseases for half a year; the absence of infectious diseases or contact with infected persons for at least two months. The uptake of peripheral blood was performed in clinical conditions using Vacutainer vials (Becton Dickinson and Company, Plymouth, UK) treated with heparin sodium (17 µ/mL).

### 2.1. Reagents

PBS (pH 7.4); RPMI 1640 medium tested for the absence of endotoxins, containing 25 mM HEPES, NaHCO_3_, and L-glutamine; and LPS *Escherichia coli* 055:B5 were purchased from Sigma-Aldrich (St. Louis, MO, USA). Alexa Fluor 488 Anti-Human CD284 (TLR4), Clone HTA125 was purchased from eBioscience (San Diego, CA, USA). RBS Lysis Buffer, Cell Staining Buffer; Alexa Fluor 488 Anti-Human CD14, Clone HCD14; Alexa Fluor 488 Anti-Human CD11b, Clone ICRF44; Alexa Fluor 488 Mouse IgG1, k isotype Crtl (FC), Clone MOPC-21; and Alexa Fluor 488 Mouse IgG2a, k isotype Crtl, Clone MOPC-173 were purchased from BioLegend (San Diego, CA, USA). The recombinant protein Der p 2 was purchased from MyBioSource (San Diego, CA, USA). Cytokines and chemokines were determined using Human ELISA Kits for TNF-α, IL-1β, IFN-γ, and IL-8 from JSC VECTOR-BEST (Novosibirsk, Russia).

Non-toxic LPS *R. capsulatus* PG (IBBP RAS) was obtained using a technique described earlier [18].

### 2.2. Surface Receptors of Monocytes and Neutrophils

For the determination of the expression of TLR4, CD14, and CD11b receptors on monocytes and neutrophils, blood (100 μL) was supplied with 5 μL of the corresponding Alexa Fluor 488-labeled mAb and incubated for 30 min at room temperature, 4 °C in the dark. The erythrocytes were disrupted using lysis buffer for 5 min at room temperature, and the leukocytes were sedimented via centrifugation (300× *g*, 5 min), washed with staining buffer, and resuspended in 400 μL of this buffer. Mouse IgG1 to mAb to CD14 and CD11b receptors and IgG2a to mAb to TLR4 served as negative controls. The expression of the receptors on the monocytes and neutrophils was estimated as the mean value of fluorescence intensity (MnI), and a gate corresponding to neutrophils or monocytes was preliminarily selected based on an FS-SS histogram. The samples were analyzed on a flow cytometer, EPICS XL-MCL (Beckman Coulter, Brea, CA, USA). At least 6000 events per sample were counted.

### 2.3. Activation of the Blood Cells by LPS, the Allergen, or Their Combination

To study the effect of LPS, the allergen, or their combination on the expression of surface receptors on monocytes and neutrophils, blood (100 μL) was incubated with LPS *E. coli* (100 ng/mL), Der p 2 (10 μg/mL), or their combination for 2 h at 37 °C in 5% CO_2_.

To study the effect of LPS, the allergen, or their combination on the synthesis of cytokines and chemokines, blood (100 μL) was diluted with RPMI 1640 medium at a 1:10 ratio and incubated with LPS *E. coli* (100 ng/mL), LPS *R. capsulatus* PG (1000 ng/mL), Der p 2 (10 μg/mL), or their combination for 6 and 24 h at 37 °C in 5% CO_2_. The samples without the addition of activating substances served as a control. To determine the antagonistic effect of LPS *R. capsulatus* PG, blood was incubated for 30 min with LPS *R. capsulatus* PG (1000 ng/mL); then, LPS *E. coli* (100 ng/mL), Der p 2 (10 μg/mL), or their combination was added and incubated for 6 and 24 h at 37 °C in 5% CO_2_. After incubation, the blood cells were centrifugated (300× *g*, 10 min) in a centrifuge, LMC-3000 (Biosan, Latvia). The supernatants were collected and stored at –20 °C until the determination of cytokine and chemokine content.

### 2.4. Cytokine and Chemokine Content

Cytokine and chemokine content was measured by using commercially available ELISA kits according to the manufacturer’s protocols. Optical density was measured using an ELISA analyzer STAT FAX 3200 (Awareness Technology Inc., Palm City, FL, USA) at a 450 nm wavelength. Quantitative estimation of the results was provided by a calibration curve reflecting the dependence of the optical density on the concentration of protein to be analyzed.

### 2.5. Data Analysis

The results are presented as medians (Me) with an interquartile range (IQR). The reliability of differences between the median values was determined using the Mann–Whitney U-test. A value of *p* < 0.05 was considered statistically significant for differences in medians. Microsoft Office Excel 2010 (AtteStat plugin), STATISTICA 10.1, and the OriginPro 7.5 program packages were used for statistical analysis and data representation. The results of flow cytometry were processed in the Flowing Software v 2.5 program package (Perttu Terho, Turku Centre for Biotechnology, Turku, Finland).

## 3. Results

### 3.1. Expression of TLR4, CD14, and CD11b Receptors on the Surface of Monocytes and Neutrophils

One of the manifestations of the biological activity of pro-inflammatory agents, including endotoxins, is the alteration of receptor expression on the surface of immune cells [19]. To compare the biological activities of LPS *E. coli*, Der p 2 allergen, and their combination, we determined the alteration of the levels of TLR4, CD14, and CD11b expression in the monocytes and neutrophils of human whole blood as a result of stimulation with these pro-inflammatory agents.

**Figure 1:**
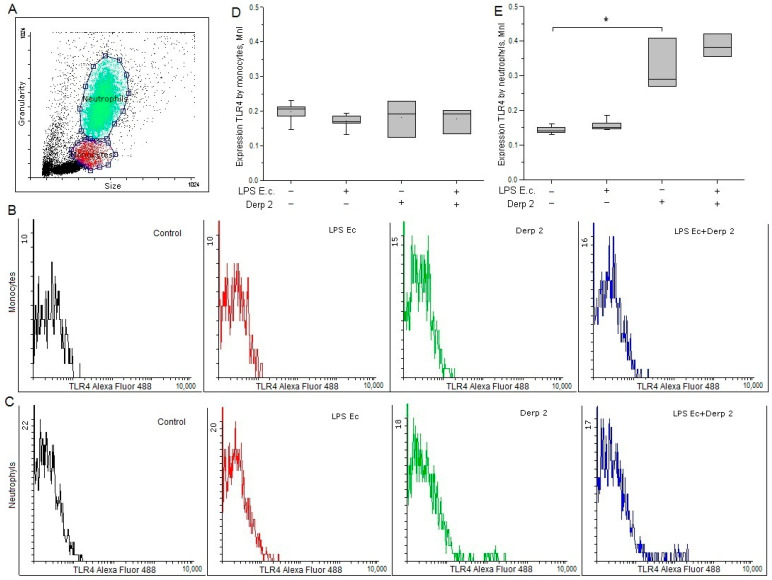
Expression of TLR4 in the monocytes (**B**,**D**) and neutrophils (**C**,**E**) of whole human blood after activation with LPS *E. coli*, Der p 2, or their combination (*n* = 5). (**A**) The dot plot shows the gating for monocytes (red gate) and neutrophils (green gate). * *p* < 0.05.

The expression of TLR4 on the surface of the monocytes was reduced upon the activation of the blood cells by LPS *E. coli*. In response to the Der p 2 allergen expression of TLR4 on monocytes did not alter significantly (Figure 1B,D). LPS *E. coli* did not affect the surface expression of TLR4 on neutrophils, while Der p 2 remarkably enhanced the expression of TLR4 on the surface of these cells (Figure 1C,E).

**Figure 2:**
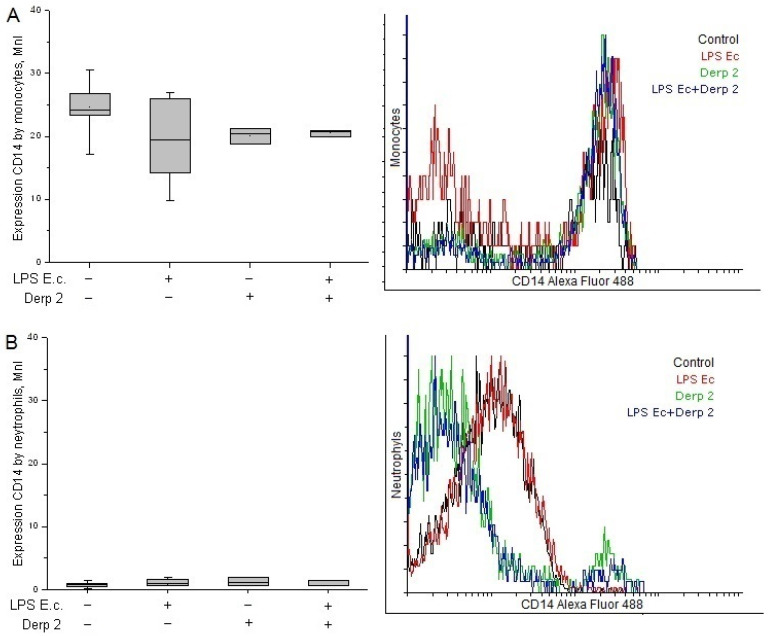
Expression of CD14 in the monocytes (**A**) and neutrophils (**B**) of whole human blood after activation with LPS *E. coli*, Der p 2, or their combination (*n* = 5).

The expression of CD14 on the surface of the monocytes was reduced upon the activation of the blood cells caused by LPS *E. coli* (Figure 2A). The Der p 2 allergen and its simultaneous action with LPS *E. coli* alleviated the expression of CD14 caused by the monocytes compared with the control. Contrary to the monocytes, the neutrophils had a low surface level of CD14 receptors and did not reliably change upon activation with the studied agents (Figure 2B).

**Figure 3:**
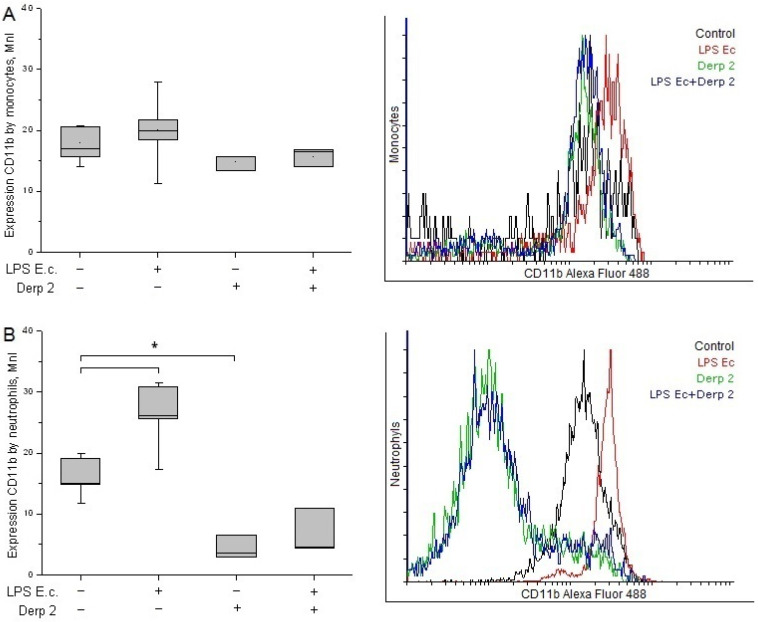
Expression of CD11b in the monocytes (**A**) and neutrophils (**B**) of whole human blood after activation with LPS *E. coli*, Der p 2, or their combination (*n* = 5). * *p* < 0.05.

The expression of CD11b in the monocytes did not change significantly upon the promotion of blood cells with LPS *E. coli* and/or Der p 2 (Figure 3A). The activation of neutrophils with LPS *E. coli* caused significant enhancement in CD11b compared with the control. These results are in agreement with the literature [20]. The Der p 2 protein and its combination with LPS *E. coli* caused a notable decrease in CD11b expression in the neutrophils compared with the control (Figure 3B).

### 3.2. Protective Effect of LPS R. capsulatus PG against Allergen- and/or LPS-Induced Cytokine Secretion

The protective effect of non-toxic LPS *R. capsulatus* PG during the activation of human whole blood cells with LPS *E. coli* in combination with the Der p 2 allergen leading to the synthesis of cytokines and chemokines was investigated in the next series of experiments.

**Figure 4:**
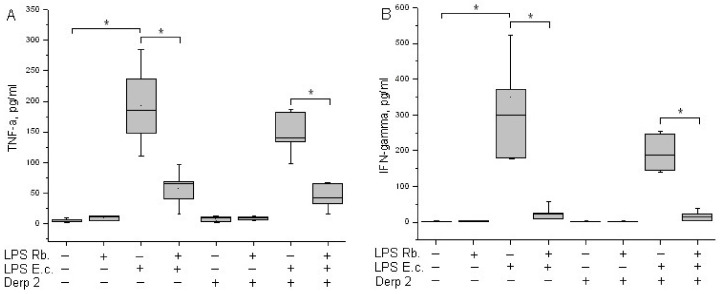
Production of TNF-α (**A**) and IFN-γ (**B**) after activation of human whole blood cells with LPS *E. coli*, LPS *R. capsulatus* PG, Der p 2, or their combination (*n* = 5). * *p* < 0.05.

TNF-α is an early cytokine produced by blood cells in response to endotoxins [21]. Cytokine TNF-α is not detected in the blood of healthy donors. LPS *E. coli* was a strong inducer of TNF-α synthesis via whole-blood leukocytes, whereas Der p 2 had low activity in TNF-α synthesis induction (Figure 4A). A combination of Der p 2 and *E. coli* endotoxin led to the alleviation of TNF-α secretion. LPS *R. capsulatus* PG did not induce the synthesis of TNF-α. The pretreatment of whole blood cells with LPS *R. capsulatus* PG decreased the TNF-α secretion induced by both LPS *E. coli* alone and its combination with the Der p 2 allergen.

IFN-γ acts as a link between innate and acquired immunity. The population of CD14+ monocytes in human blood produces IFN-γ upon activation with LPS [22]. LPS *E. coli* was a strong inducer of IFN-γ synthesis via whole-blood leukocytes (Figure 4B). Der p 2 did not activate blood cells to IFN-γ synthesis, and in combination with LPS *E. coli*, it reduced its production compared with the activation of LPS *E. coli* alone. We did not detect the activation of IFN-γ released in response to LPS *R. capsulatus* PG. The pretreatment of whole blood cells with LPS *R. capsulatus* PG significantly decreased the secretion of IFN-γ induced by LPS *E. coli* or by a combination of LPS *E. coli* and the allergen.

**Figure 5:**
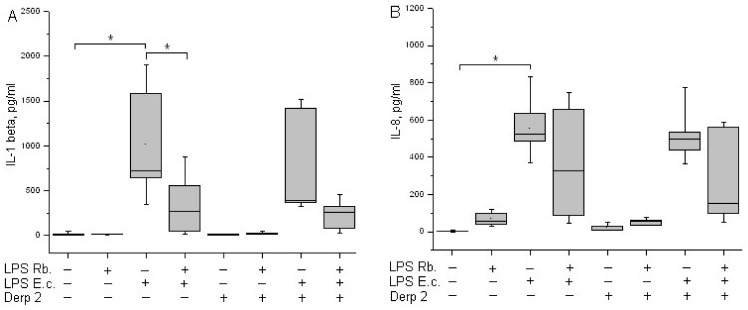
Production of IL-1β (**A**) and IL-8 (**B**) after activation of human whole blood cells with LPS *E. coli*, LPS *R. capsulatus* PG, Der p 2, or their combination (*n* = 5). * *p* < 0.05.

It was shown earlier that isolated monocytes secret IL-1β under stimulation with LPS [23]. In our experiments, the activation of whole-blood leukocytes with LPS *E. coli* led to a significant induction of IL-1β synthesis (Figure 5A). The synthesis of IL-1β in response to a combination of Der p 2 and LPS *E. coli* was comparable to the response of the cells to LPS *E. coli* alone. Der p 2 and LPS *R. capsulatus* PG did not induce IL-1β secretion. The pretreatment of whole blood cells with LPS *R. capsulatus* PG decreased the secretion of IL-1β induced by LPS *E. coli* or a combination of LPS *E. coli* and the allergen.

IL-8 is produced by many cell types, including monocytes. IL-8 is a key chemokine of neutrophils, responsible for their recruitment, survival, and inflammation [24]. A background level of IL-8 might be present in the blood of healthy donors. LPS *E. coli* is a strong inducer of the synthesis of IL-8 chemokine via whole-blood leukocytes (Figure 5B). The Der p 2 allergen did not cause blood cells to begin IL-8 synthesis. The combined activation via Der p 2 and LPS *E. coli* was comparable to the action of LPS *E. coli* alone. LPS *R. capsulatus* PG virtually did not induce the synthesis of the IL-8 chemokine. In contrast with the suppression of the synthesis of TNF-α, IL-1β, and IFN-γ, LPS *R. capsulatus* PG did not block the LPS- and/or allergen-induced secretion of IL-8.

## 4. Discussion

Widespread allergic asthma attracts the attention of scientists to the reasons for its exacerbation. One of the causes of the additional activation of the innate immune system is the contamination of allergens with endotoxins [25]. We did not find data on the simultaneous activation of blood cells with the allergen or endotoxin. In this connection, we studied the responses of healthy donor blood to the Der p 2 allergen and its combined action with LPS. The contamination of allergens with endotoxins and the ability of Der p 2 to interact with LPS demonstrate the mutual influence of the allergens and bacterial infections during inflammation progression.

The main signs of the activation of immune cells with pro-inflammatory agents are changes in cell surface receptor expression and the release of cytokines and chemokines by blood cells [19,21]. The alteration of the expression of TLR4, CD11b, and CD14 receptors on the surface of human whole blood cells under the action of LPS *E. coli*, Der p 2 allergen, or their combination reflects the different functional activities of monocytes and neutrophils. Our studies of the influence of Der p 2 on TLR4 expression via the innate immunity cells showed the enhanced expression of TLR4 in neutrophils. There are a number of confirmations that show that the direct interaction of allergens with TLR triggers inadequate immune responses [26]. TLR4 expression controls the reaction of neutrophils to inhaled LPS and allergens. Inside the network of the pathogenetic mechanisms of airway inflammation, neutrophils are considered effector cells, performing such immune functions as the detection, recognition, and elimination of bacteria and other pathogens. A study of the role of neutrophils in the progression of severe forms of asthma led to the finding that the stimulation of TLR4 in neutrophils with HDM regulates their apoptosis [27].

LPS *E. coli* and/or Der p 2 decreased the expression of TLR4 and CD14 in monocytes, which could be mediated by the CD14-dependent endocytosis of these receptors [28].

β2-integrins are expressed at high levels in monocytes. In monocyte-derived dendritic cells, it was shown that anti-CD18 mAb strongly inhibited Der p 2 responses [29]. CD11b (Mac-1, CR3) is a neutrophil surface antigen and an α-subunit of β2 integrin. On the surface of non-activated neutrophils, CD11b is expressed at a very low concentration. CD11b expression rises on the neutrophil surface after 5 min of exposure to bacteria or endotoxins, and this is a marker of activation [30]. In vitro studies showed that the stimulation of neutrophils with LPS induces the significant augmentation of CD11b levels on the cell surface and the release of IL-8 [20]. CD11b facilitates the binding of LPS to TLR4 in cell type-specific signal transduction [31].

The results of our studies on receptor expression reflect the differences in the mechanisms of activation of neutrophils and monocytes in the responses of cells to LPS and allergens associated with the specific participation of these cells in the development of inflammatory responses.

The leading activators of immune responses and major producers of pro-inflammatory cytokines are the monocytes of peripheral blood [21,32]. TLR4-signaling induced by lipid A *E. coli* leads to the synthesis of large numbers of pro-inflammatory cytokines” TNF-α, IFN-γ, and IL-12 p40 [33]. Der p 2, similar to LPS, also induces TLR4 signaling. The cell response to Der p 2 triggers the activation of the NF-κB and MAPK signaling pathways, which regulate the synthesis of inflammation intermediators in different manners [34]. Model studies on THP-1 cell cultures [3], as well as on isolated neutrophils and B-lymphocytes [12], showed that Der p 2 effectively promotes isolated cells to produce IL-6, IL-1, IL-8, CXCL10, and TNF-α. The isolated B-cells of blood also react to the allergen Der p 2 via the production of pro-inflammatory cytokines [35]. Our earlier studies on the isolated mononuclear cells of peripheral blood (PBMC) showed that, upon their activation with the antigen, PI3K-dependent signal transduction is one of the most prominent participants in the regulation of cytokine synthesis. The PI3K-dependent signal transduction system negatively regulates, for example, the production of TNF-α during the activation of cells with Der p 2, but it does not participate in the co-activation of LPS and Der p 2 [13]. Thus, the activity of the Der p 2 allergen in ex vivo experiments on whole blood samples is significantly lower compared with its activity in vitro in isolated PBMC cells, which should be taken into account when transferring the results obtained from isolated cells to whole blood cells.

TNF-α is one of the earliest cytokines produced in response to endotoxins, and it is of basic proteins associated with asthma in various populations [36]. Increased concentrations of TNF-α are found in people with different types of asthma after contact with allergens [37]. It is important to know that immune cells express of receptors for TNF-α, which are further involved with the synthesis of later pro-inflammatory cytokines such as IFN-γ and IL-1β [38].

Interferons play an important role in innate cytokine responses to inflammation, thus providing immunomodulating activity [39]. Upon the activation of monocytes and macrophages with toxic LPS, elevated levels of IFN-γ mRNA were demonstrated. The expression of MD-2 and MyD88 also increased [40]. In our experiments, LPS *E. coli* was a strong inducer of IFN-γ (Figure 4B).

Pro-inflammatory cytokine IL-1β is a key mediator in infectious, autoimmune, and allergic diseases [41]. In our study, LPS *E. coli* showed the significant activation of IL-1β via leukocytes, whereas Der p 2 affected the process of cytokine synthesis very slightly (Figure 5A).

IL-8 is a key chemokine of neutrophils responsible for recruitment, survival, and inflammation, and it plays an important role in the pathogenesis of airways during asthma [24]. We have shown that the simultaneous activation of blood cells with LPS *E. coli* and the Der p 2 allergen had virtually no effect on the induction of IL-1β and IL-8 compared with activation using LPS *E. coli* alone (Figure 5B).

Our study on whole blood showed that Der p 2 (10 μg/mL) weakly activates blood cells to produce pro-inflammatory cytokines (Figure 2). The experimentally observed low activity of Der p 2 could be conditioned by the fact that, when it enters the blood, this allergen displays the properties of a cholesterol binder rather than an MD-2 mimicking agent in the circulating blood [42]. Other blood proteins, in particular, heparin, also bind Der p 2 and block its activity [43]. The presence of IgE in the donors’ blood cannot be excluded, and this protein specifically binds Der p 2, thus decreasing its pro-inflammatory activity [44].

We confirmed in our earlier and current experiments that non-toxic LPS *R. capsulatus* PG did not activate blood cells to synthesize TNF-α, IL-1β, IFN-γ, or IL-8 when taken in concentrations exceeding the concentration of *E. coli* endotoxin by tenfold [16,17]. The pre-incubation of whole blood cells with LPS *R. capsulatus* PG decreased the secretion of TNF-α, IL-1β, and IFN-γ induced by LPS *E. coli*. The results showing the absence of the protective effect of LPS *R. capsulatus* PG from IL-8 chemokines released by blood cells could be explained by more complicated mechanisms of regulation in this chemokine synthesis, which enrolls not just TLR4 and CD14 but also extra receptors in LPS, for example, TREM1, which is expressed in myeloid cells [45,46].

In our experiments. the co-activation of blood cells with LPS *E. coli* and Der p 2 decreased the synthesis of the MyD88-dependent pro-inflammatory cytokines TNF-α and IFN-γ (Figure 4) and had almost zero effect on the synthesis of IL-1β or IL-8 compared with activation via the endotoxin alone (Figure 5). LPS *R. capsulatus* PG blocked the synthesis of the rapid cytokine TNF-α and the late cytokines IL-1β and IFN-γ by blood cells in response to simultaneous activation via LPS *E. coli* and Der p 2, which confirms the competition between these two agents for TLR4.

LPS *R. capsulatus* PG remarkably decreases the synthesis of cytokines activated by toxic LPS, the allergen, or their combination. This indirectly indicates the common mechanisms of the induction upon the activation by endotoxins and allergens and the common mechanism of the protective action of LPS *R. capsulatus* PG against both the endotoxins and its simultaneous action with the allergen.

Currently, it is unknown whether Der p 2 is a sufficient stimulus or just an adjuvant promoting the activation of the innate immunity of cells in whole blood caused by endotoxins. The results of this work provide a better understanding of the mechanisms of the simultaneous activation of blood cells caused by the different exogenous factors: LPS and Der p 2.

## 5. Conclusions

The obtained data on the expression of TLR4, CD14, and CD11b receptors in human whole blood monocytes and neutrophils in response to LPS *E. coli*, the Der p 2 allergen, or their combination reflect the different functional activities of these cells, while the composition and number of produced cytokines reflect the biological activity of the studied agonists. Our results indicate that the activity of the Der p 2 allergen in ex vivo experiments on whole blood samples is significantly lower compared with its activity in vitro in isolated PBMC cells. This may be due to the peculiarity of our experimental model, in which blood proteins could specifically bind to the allergen and reduce its pro-inflammatory activity. LPS *R. capsulatus* PG significantly decreases the synthesis of MyD88-dependent, NF-κB-regulated cytokines activated by LPS *E. coli*, Der p 2, or their combination. This indirectly indicates the general mechanism of cell activation via these structures and the unified mechanism of the protective action of LPS *R. capsulatus* PG against both endotoxins and an endotoxin and allergen combination.

## Data Availability

The data presented in this study are available upon request from the corresponding author. The data are not publicly available for ethical reasons.
